# Charge to the Graduates in Dentistry

**Published:** 1889-05

**Authors:** 


### CHARGE TO THE GRADUATES IN DENTISTRY.

To you young gentlemen of the Dental class, I need add but few remarks. The principles I have commended to the graduates in medicine apply with equal force to yourselves.

We are pleased to have had you in our Institution during this session, and congratulate you as graduates in the Dental Department of the Southern Medical College.

This branch of our school was established for the benefit and convenience of Southern young men especially, who should choose the profession of Dental Surgery, and for whom we have provided needed facilities in that department. To those who from any other section desire dental instruction in a mild climate with healthful surroundings, we can assure them they can find here all that is requisite, and that they will be made to feel at home among friends.

Every section of our common country should be self-sustaining in its progressive enterprise, and make available every resource offered by nature and opportunity, not only in minerals, agricultural and manufacturing development, but in the educational interest, so important to the rising generation, and so essential to the destiny and permanency of a great nation.

It is the duty of every man to stand by his own people and section, and lend a helping hand to uphold his own corner in whatever place his lot is cast. But in giving aid to ourselves, we need not and should not detract from the interest of other sections, and the development of their resources, nor will it prevent us from rejoicing in their prosperity. This is true patriotism, and the true paternal spirit.

Though the fortunes of war did not permit us to "divide the house," we as Southerners can labor to improve and make secure our corner of the common building. The more independent we become in our own section, the more we will be respected by the people of other parts of our great country.

The South is deserving of restitution; that would be a simple act of justice; but, though it may never be given to us, we, as her people can, and should uphold her best interests, give our hearts, hands, brains and money to advance the great possibilities of her future prosperity, and with one mind, stand shoulder to shoulder in undeviating loyalty to what is best and greatest for the loved land of our birth.

We heartily welcome all noble men and women of every clime who come among us, especially those who appreciate our condition and feelings; who identify themselves with us, and who leaving behind them all sectional antipathies and prejudices, recognize our equality in the union as men and patriots, and co-operate with us in rebuilding the waste places and developing the resources of our Section.

Yet upon Southerners at last, rest the greatness of the mission and the obligation to build up the beautiful and solid proportions of the future South that will command the respect and admiration of both friends and foes.

To do this, Southerners must patronize home institutions, encourage home talent in every art, in science, education, literature, mechanics, commerce and agriculture, and make all legitimate means and methods bring rich tribute to lay at the feet of our own beloved South.

This is not selfish nor ungenerous, but is the fair-minded and truly patriotic spirit which should inspire the hearts and deeds of the people of every section. And my young friends, I would have you take the animus of these principles out with you into the world in your profession and your life, and inculcate them in the minds of your associates and fellow citizens.

The Dental Department of the Southern Medical College, as has been said, was established for the benefit and convenience of our young men, and as its friends have endeavored to make it in every respect worthy of patronage, it is but fair that it should receive the encouragement it has tried to deserve.

To this, our second graduating class in Dental Surgery, we give our cordial commendation, and thank them for their studious application and the respectful attention they have given their kind, noble and learned instructors during the term just closed.

Young gentlemen, we wish you increasing and deserved success in your profession, and beg you not to be satisfied with less than the best knowledge and highest skill in Dental Surgery.

Be true to yourselves and faithful to your obligations; be true to your manhood and all noble principles; be true to Alma Mater and remember, "He who lives well, dies well" is a maxim to be treasured in memory. Let it be the rule of your conduct, and may Heaven's richest blessings go with you through life.

